# Comparing effectiveness of mineral trioxide aggregate, bioceramic putty and tannic acid in maintaining pulp vitality after experimental pulpotomy in rats

**DOI:** 10.1038/s41598-024-80601-0

**Published:** 2024-11-27

**Authors:** Aya Anwar Alsherif, Mohamed Salah, Mai Badreldin Helal

**Affiliations:** Faculty of Dentistry, Tanta University, El-Giesh St., Tanta, Gharbia Egypt

**Keywords:** Drug discovery, Health care, Medical research

## Abstract

This study aimed to evaluate and compare the effectiveness of mineral trioxide aggregate (MTA), bioceramic putty (BP) and tannic acid (TA) for experimental pulpotomy. Our in-vivo experimental study involved sample of 45 rats that were randomly divided into 4 groups: Group 1 (subdivided into negative (1-A) and positive (1-B) subgroups), Group 2 (MTA treated), Group 3 (BP treated) and Group 4 (TA treated). 4 weeks post pulpotomy, specimens were analyzed histologically, immunohistochemically using dentin sialoprotein marker, and histomorphometrically by assessing the thickness of newly formed dentin bridge. Group 1-B showed pulp necrosis without hard tissue formation. Group 2 showed moderate dentin formation while group 3 presented a thick layer of calcific barrier. Group 4 showed dentin bridge formation, however, irregular pulp calcifications and radicular pulp necrosis were seen. The thickness of newly formed dentin bridge showed a significant difference between group 1-B and group 2, 3 &4. Significant difference was found between group 2&3 and group 3&4. Dentin sialoprotein immunohistochemical expression was negative in group 1-B, mild in group 2, strong in group 3 and moderate in group 4. MTA and BP proved to be effective pulpotomy agents with BP being superior. For TA, further studies are required to explain the recorded unfavorable effects in some specimens.

## Introduction

Carious deciduous molars with coronally inflamed vital pulp are a frequently encountered dental issue in young patients. Early extraction of such teeth can dramatically affect arch length, esthetics, mastication, speech, and development of abnormal habits. Fortunately, this can be retrieved by pulpotomy, a vital pulp therapy technique that aims to preserve the healthy radicular part of pulp tissue that is supposed to be capable of healing after surgical amputation of the affected coronal part^1^.

Numerous remedies with several protocols had been documented to maintain radicular pulp vitality. It was proved that the success of any pulpotomy protocol depends mainly on the type and properties of medicament used. Mineral trioxide aggregate (MTA) was introduced as pulp regenerative material and now is known as the reference material for vital pulp therapy with outstanding success rates (90–100%)^2^. Minimal pulpal inflammation with remarkable deposition of reparative dentin was primarily noticed in MTA treated pulp tissues^3^. However, MTA has some serious limitations including difficult handling and containing heavy metals like alumina and bismuth oxide^4^.

Recently, High-tech improvements in the curative materials headed to invention of bioceramics that display exceptional biocompatibility. Bioceramic putty (BP), an insoluble, radiopaque calcium silicatebased material with low cytotoxicity, is presented as a rootrepairing material which stimulates hydroxyapatite deposition on its surface when exposed to tissue fluids^5^. It is a newly developed bioactive material served as a ready-to-use white premix in putty form. BP not only displays reasonable biocompatibility, sealing capacity, and antibacterial activity but also enhances the expression of mineralization-related genes. It is also considered to be superior to MTA owing to its easy handling, better physical properties, high viscosity and shorter setting time^5–7^.

Tannic Acid (TA) is a naturally occurring polyphenol that can be extracted from oak tree galls. Classically, it has been used in various historical applications including leather tanning, iron gall ink and red wines^8^. Over the past decade, TA had gained attention in the biomedical researches for its unique biochemical characteristics that mainly depend on having many hydroxyl groups that are favorable to many molecular interactions with different biomolecules^9^. Interestingly, TA has shown to have a strong anti-inflammatory, antioxidant, antiviral, antifungal and antibacterial activity^10^. Altogether, TA provides a vast range of potential applications that can be crucial to various biomedical and biomaterial researches.

Hence, this experimental study was designed to evaluate and compare the in vivo effectiveness of MTA, BP and TA for experimental pulpotomy in rats.

## Materials and methods

### Study design

The authors complied with the ARRIVE guidelines. This study was conducted as in vivo experimental study that was designed in accordance with the guidelines of the scientific research ethics recommendation of Ethical Committee at Faculty of Dentistry, Tanta University, Egypt. The experimental protocol was approved by the committee with an ethical approval # R-OB-3-23-3.

### Animals

A sample of 45 male Wistar albino rats of 8-weeks old, weighing between 180 and 200 g, were purchased from the animal housing unit of Histology department, Faculty of medicine, Tanta University and incorporated in this study. The rats were housed in individual cages, 2 per cage, kept in a room with a controlled temperature of 22 ± 1 °C and a 12 h light\dark cycle, with free access to standard water and food ad-libitum.

The sample size and power analysis were calculated via Epi-Info software statistical package generated by World Health organization and center for Disease Control and Prevention, Atlanta, Georgia, USA version 2002. The standards used for sample size calculation were as follows: 95% confidence limit and 88% power.

### Experimental procedures

#### Pulpotomy procedure

First, rats were anesthetized according to *Kawai et al.*^11^ protocol using a combination anesthetic including: 0.3 mg/kg of medetomidine, 4.0 mg/kg of midazolam, and 5.0 mg/kg of butorphanol. The rats were pinned on their back to a surgical board with the right maxillary first molars being disinfected with 5.25% sodium hypochlorite. Then, with the aid of a dental surgical loupe, the pulp chamber was mechanically exposed occlusally using high-speed handpiece with sterile 0.6-mm diameter round diamond bur under continuous water cooling. Coronal pulp was excavated, and the pulp chamber was washed by sterile saline. Hemorrhage was controlled by applying light pressure with wet cotton pellets followed by irrigation with sterile saline^12^. After hemorrhage control, the corresponding pulpotomy material was used according to each group.

#### Animals grouping

Rats were randomly assigned, using block randomization technique, into 4 groups as follows:

- *Group 1 (n = 15)*: this group was subdivided into:
  1. Subgroup A (negative control group, n = 5): in which rats didn’t receive pulpotomy.
  2. Subgroup B (positive control group, *n* = 10): in which, rats received pulpotomy but left without being capped by any pulpotomy agent.
- *Group 2 (MTA group, n = 10)*: after completion of pulpotomy procedures, MTA (BIO MTA, 37–450 Stalowa Wola, Poland) was prepared according to manufacturer’s recommendation. 1 g of MTA powder was mixed with 1 vial of distilled water to get a smooth mix that was directly applied over the pulp stump.
- *Group 3 (BP group, n = 10)*: after completion of pulpotomy procedures, BP (Well-Root PT, VERICOM Co., Ltd.), premixed according to manufacturer’s instructions, was applied directly over the pulp stump.
- *Group 4 (TA group, n = 10)*: after completion of pulpotomy procedures, TA was mixed with distilled water to get a smooth mix that was directly applied over the exposed pulp tissue.

Finally, all cavities of the study groups were coronally sealed with light-cured glass ionomer cement, followed by self-adhering flowable composite resin restoration^13^.

#### Histological analysis

After 4 weeks, all rats were euthanized, using an overdose of ketamine and xylazine^14^, and the maxillary first molar area was dissected and fixed in 4% paraformaldehyde for 24 h at 4 °C. The specimens were demineralized in 10% EDTA/phosphate buffered saline solution and then dehydrated and embedded in paraffin blocks. Mesiodistal sections, with a thickness of 5 μm, were cut and prepared for *hematoxylin-eosin* staining according to the manufacturer’s protocol. The sections were examined under light microscope (Olympus America Inc.) and images were captured using Light Microscope built in camera (LEICA ICC50 HD Camera system) via image software LAS EZ version 3.0.0. An assessment of the severity of the inflammatory infiltrate was done according to the degree of immune cells infiltration into the target tissue. It was expressed as no inflammation, mild, moderate or severe inflammation.

#### Statistical analysis

Using images of hematoxylin-eosin stained sections captured under a standard magnification x 400, the thickness of newly formed dentin bridge was assessed, by a blinded examiner, in all experimental groups’ specimens according to the following grading^15^.

- Grade 1: Heavy hard tissue deposition as a complete dentin bridge (each specimen equals 4 points).
- Grade 2: Moderate hard tissue deposition (each specimen equals 3 points).
- Grade 3: Only a slight layer of hard tissue deposition (each specimen equals 2 points).
- Grade 4: No hard tissue deposition (each specimen equals 1 point).

The score of each group was calculated and the quantitative data were statistically analyzed using CO-STAT analysis (version 6.4). Numerical variables were expressed by descriptive statistics as mean, standard deviation and range. One way ANOVA and post hock test (tukey-test) were used to compare quantitative data between groups.

#### Immunohistochemistry

For immunohistochemical analysis, dentin sialoprotein (DSP) staining was performed to investigate odontoblastic activity and dentin formation. Sections of 5 μm thickness were deparaffinized, rehydrated, and rinsed with distilled water. Protease K (Dako, Carpinteria, CA) was utilized for antigen retrieval. Endogenous peroxidase was deactivated by adding 3% hydrogen peroxide. Then, sections were incubated in 5% bovine serum albumin (Sigma-Aldrich) incubated with primary antibody all night. A 1:500 dilution of the anti-DSP antibody was used. The sections were counterstained with hematoxylin stain^16,17^.

## Results

### Histological results

#### Hematoxylin-eosin staining

**Fig. 1:**
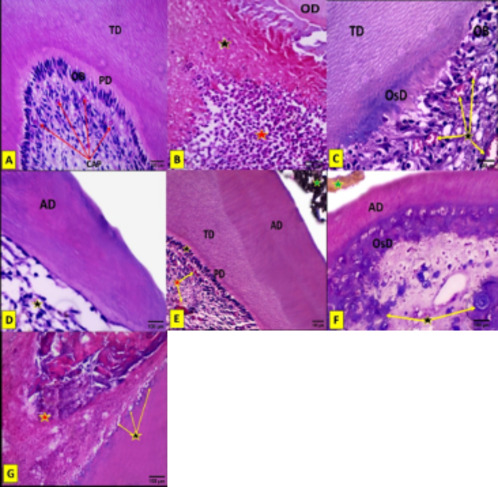
Photo micrographs demonstrating light microscopic evaluation of study groups. (**A**) Group 1-A showing normal pulp zones with distinct regularly arranged odontoblastic layer (OB) separated from tubular dentin (TD) by a well-defined predentin layer (PD). The pulpal capillaries (CAP) demonstrated no congestion. (**B**) Group 1-B showing huge areas of dead pulp tissue (black asterisk) with a moderate to severe inflammatory reaction in the radicular pulp (red asterisk). OD: old dentin. C& D: Group 2; (**C**) Showing formation of a thick layer of tubular dentin (TD) as well as areas of osteodentin (OsD). No definite odontoblastic zone (OB) or predentin layer was detected. Moderate pulpal capillary congestion (arrows) was seen. (**D**) Another specimen showed formation of a thinner layer of atubular dentin (AD) and indefinite pulp zones (asterisk). (**E**) Group 3 showing a thick layer of newly formed calcific barrier in the form of a layer of atubular dentin (AD) followed by tubular dentin (TD). Distinctive pulp zones with mild inflammatory infiltration and moderate capillary congestion (red asterisk) were seen. PD: predentin, black asterisk: distinct odontoblast-like cells, green asterisk: remaining BP material. F& G: group 4; (**F**) Showing dentin bridge formation (atubular dentin (AD) followed by osteodentin (OsD)). Irregular pulp calcifications were seen (arrows). No definite pulp zones could be detected. Green asterisk: remaining TA material. (**G**) In one specimen, radicular dentin resorption (arrows) and pulp necrosis (red asterisk) were seen. (H&E stain, original magnification ×400).

*Group 1-A*:

Teeth of the negative control group were examined to provide comparison between untreated and treated pulp tissues. Upon group I-A examination, normal pulp zones were noticed with distinct regularly arranged odontoblastic layer separated from tubular dentin by a well-defined predentin layer. The pulpal capillaries demonstrated no congestion. No inflammation or abnormal pulp calcifications were observed (Fig. 1A).

*Group 1-B*:

All specimens of the positive control group exhibited huge areas of dead pulp tissue. Pulpal necrosis extended from the site of pulp exposure to include nearly the whole pulp chamber. Also, the specimens showed a moderate to severe inflammatory reaction in the radicular pulp. No hard tissue formation was observed at the exposure site (Fig. 1B).

*Group 2*:

In MTA treated group, a moderately thick layer of tubular dentin was formed, however, areas of osteodentin were also seen. No definite odontoblastic zone or predentin layer was detected. Mild inflammatory infiltration and moderate pulpal capillary congestion were seen. Additionally, in three specimens, a thinner layer of atubular dentin was formed and indefinite pulp zones were detected (Fig. 1C,D).

*Group 3*:

In BP treated group, a thick layer of calcific barrier was formed, composing of a layer of atubular dentin followed by tubular dentin. A definite predentin layer separated the dentin bridge from a distinct layer of newly formed odontoblast-like cells. Distinctive pulp zones with mild inflammatory infiltration and moderate capillary congestion were seen (Fig. 1E).

*Group 4*:

TA treated group showed dentin bridge formation, composing of atubular dentin followed by osteodentin. However, irregular pulp calcifications were also seen. No definite pulp zones could be detected. In one specimen, dentin resorption and radicular pulp necrosis were recorded (Fig. 1F,G).

#### Immunohistochemical staining

**Fig. 2:**
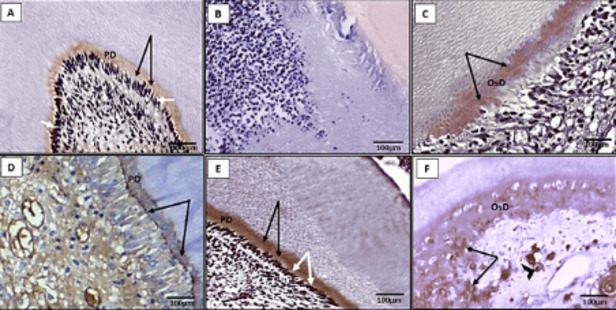
Photomicrographs displaying immunolocalization of dentin sialoprotein (DSP) in different study groups. (**A**) Group 1-A showing positive expression of DSP within the matrix of predentin layer (PD, black arrows) together with nuclear immunostaining within the odontoblast cells (white arrows). (**B**) Group 1-B shows negative immunohistochemical expression of DSP. (**C**): Group 2 shows positive mild expression of DSP within some areas of the newly formed osteodentin (OsD, black arrows). (**D**,**E**): Group 3 illustrating strong DSP reaction within the dentin-pulp complex. It involves predentin layer (PD) (black arrows) and odontoblast-like cells (white arrows). (**F**): Group 4 depicts moderate immunostaining only in discrete areas within the newly formed osteodentin layer (black arrows) together with some stromal cells (black arrowhead) (dentin sialoprotein immunostaining counterstain with Mayer’s hematoxylin., original magnification ×400).

Immunohistochemically, DSP expression was observed in *Group 1-A* in the predentin layer together with the odontoblastic layer (Fig. 2A). On the other hand, *Group 1-B* revealed negative immunohistochemical reaction for DSP in the dentin-pulp complex (Fig. 2B). *Group 2* showed positive mild expression of DSP within a thin area of the newly formed osteodentin (Fig. 2C). Interestingly, dentin-pulp complex of *Group 3* specimens portrayed strong positive immunoreactivity at the predentin layer and its lining odontoblast-like cells (Fig. 2D,E). Whereas, immunohistochemical localization for DSP in *Group 4* depicted moderate positive immunoreactivity in the newly formed osteodentin layer (Fig. 2F).

### Statistical results

**Fig. 3:**
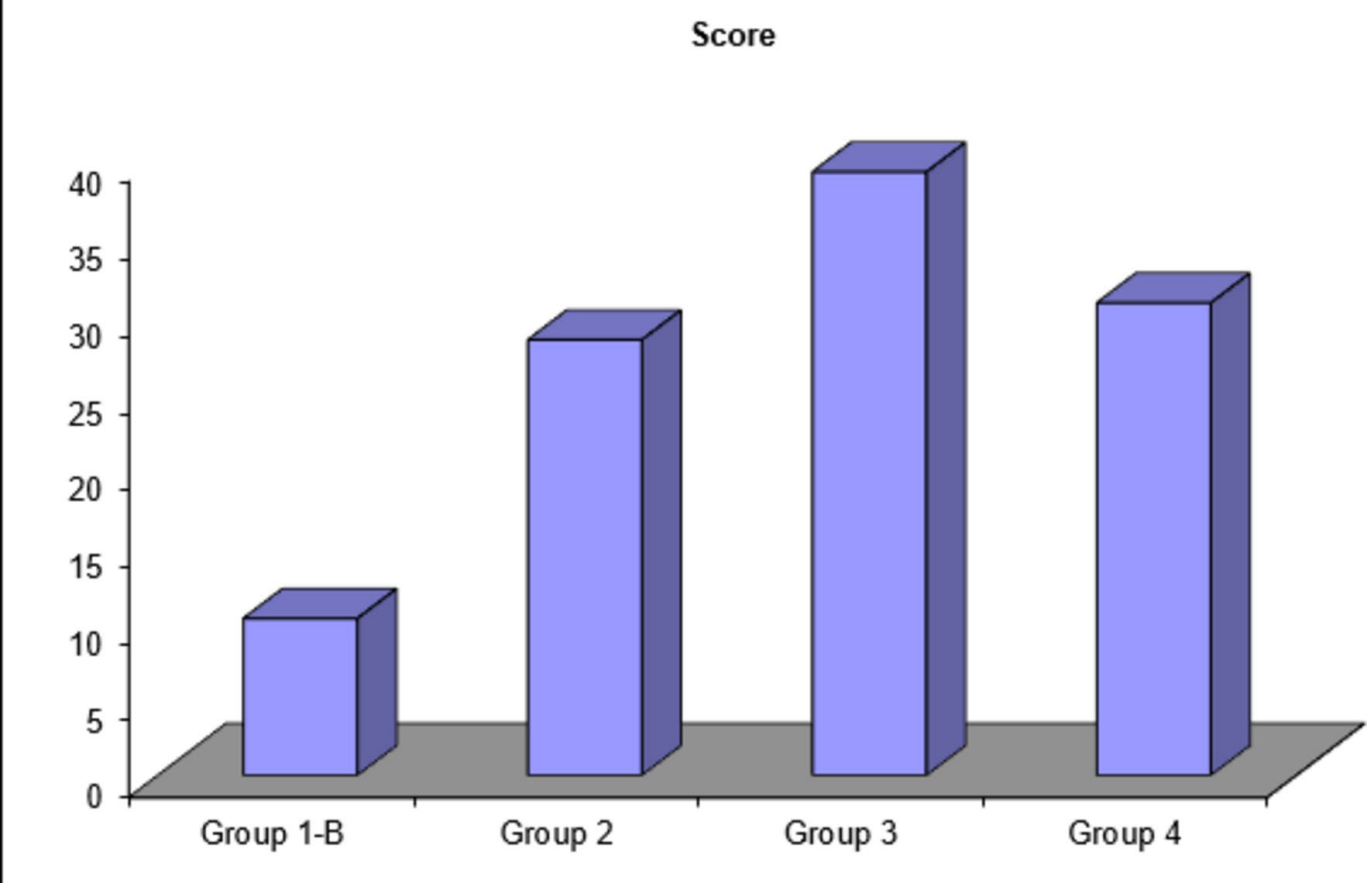
Showing statistical analysis of the thickness of newly formed dentin bridge between different groups using ANOVA and Tukey-test.

Upon assessment of the thickness of the newly formed dentin bridge of different groups, the following scores were obtained (Table 1) and were statistically analyzed. Statistical analysis between different groups revealed a significant difference between group 1-B and both of group 2, 3 &4. Also, a significant difference was found between both group 2&3 and group 3&4. However, the difference between group 2&4 was non-significant (Table 2, Fig. 3).

**Table 1:** Showing grades of hard tissue formation of specimens in different groups and the final score of each group.

	Group 1-B	Group 2	Group 3	Group 4
Grade 1	0	1	9	3
Grade 2	0	6	1	4
Grade 3	0	3	0	3
Grade 4	10	0	0	0
Score	10	28	39	30

**Table 2:** Showing statistical analysis of the thickness of newly formed dentin bridge between different groups using ANOVA and Tukey-test.

Score		Group 1-B		Group 2		Group 3		Group 4	
**Mean ± SD**		10.2 ± 2.53		28.3 ± 4.40		39.20 ± 3.85		30.7 ± 4.0	
**F test**		105.164							
**P value**		0.001*							
1-B & 2	1-B & 3		1-B & 4		2 & 3		2 & 4		3 & 4
0.001*	0.001*		0.001*		0.001*		0.162		0.001*

(*) significant difference (*P* < 0.05).

## Discussion

Pulpotomy in primary dentition has encountered significant challenges in recent years. This procedure is influenced by various factors including accurate diagnosis and technique, the operator’s efficiency, the choice of final restoration, and the most important one is the selection of pulpotomy agent material.

Over years, to achieve highest success rates of pulpotomy, vast range of agents have been applied such as formocresol, glutaraldehyde, calcium hydroxide, enamel matrix derivatives, bone matrix proteins and MTA. Moreover, different techniques as electrosurgery and laser were tried out, however, trials to find an ideal pulpotomy agent is still going on^18^.

In the present work, we have chosen rats whose dental tissues, as well as oral bacterial flora, are more comparable to that of humans than other commonly used experimental species^19^. When exposing vital pulp tissue of rats, an inflammatory response, resembling that found in humans, was detected, however, this response is faster in rats when compared to humans^20^. Previous researches admitted that reparative dentin found in rats after pulp capping was almost identical to that found in human teeth^12^. Further, rat maxillary molars were involved in this study owing to its anatomical and histological resemblance to human molar teeth^21^. Therefore, analyzing both inflammatory and healing mechanisms following experimental pulp capping in a rat model is thought to provide results representative to humans.

At the end of the pulpotomy procedure, all cavities of the study groups were coronally sealed with light-cured glass ionomer cement, followed by self-adhering flowable composite resin restoration to avoid the predictable danger of bacterial invasion which results in pulp inflammation and necrosis^13^.

Upon evaluating the histological results, specimens of the ***group 1-A*** showed normal pulp zones with distinct regularly arranged odontoblastic layer separated from tubular dentin by a well-defined predentin layer. DSP expression was typically observed in the predentin layer together with the odontoblast cells as already stated by several previous researches^22^.

When discussing ***group 1-B***, we have to explain that this group, where rats received pulpotomy but left without being capped by any pulpotomy agent, was applied following the protocol of *Kramer PR et al.*^16^. This was thought to provide a model to mimic clinical cases, where pulp exposure occurs due to caries or fracture, and study the corresponding defense pulp mechanism. Herein, the histological examination showed extensive coronal pulp necrosis with moderate to severe inflammatory reaction in the radicular pulp and no hard tissue formation that was further confirmed by negative immunohistochemical reaction for DSP in the dentin-pulp complex. This came in accordance with that found by *Kramer PR et al.*^16^ who recorded absence of viable pulp tissue in rat molars experimentally exposed then restored without pulp capping material. This can be attributed to the results recorded by the same mentioned research where proinflammatory cytokines, IL-1α and IL-1β, levels were significantly higher, after 30 days follow up period, in the rats with exposed pulps versus rats that had their pulp capped together with detection of bacteria and immune cells that can stimulate and produce these cytokines. So, lack of an effectual pulp capping material placed in direct contact with the vital pulp can explain the result attained in our positive control group.

In ***group 2***, MTA was chosen to compare the results of experimental materials to it as it had already provided a consistent high success rate of 95–100% as a pulpotomy agent^2^. Upon histological evaluation, the MTA treated specimens showed formation of a moderately thick layer of tubular, or atubular, dentin and areas of osteodentin. The DSP immunohistochemical detection showed positive mild expression of DSP within a thin area of the newly formed osteodentin. This came in agreement with *Dianat O. et al.*^23^ study that reported formation of a thick layer of dentin that closed the exposure site completely in all MTA treated rats after 30 days. Many previous studies have tried to explain how MTA enhances dental tissue repair. Some proposed production of hydroxyapatite crystals when calcium ions, released by MTA, come in contact with tissue fluids^24^. Others postulated that its reparative effect is due to a chemical injury that triggers pulpal irritation hence stimulates repair^25^. As well, the MTA alkaline pH provides a favorable environment for dentine formation^26^. It can also induce the proliferation of human pulp cells and its differentiation to odontoblast-like cells, through releasing calcium and hydroxyl ions^27^, and increase its expression of mineralization promoting genes^28^. Recently, some studies proposed that MTA may encourage dentinal repair through releasing bio-active molecules from the dentin matrix^29^.

On contrary, *Dianat O. et al.*^23^ recorded absence of inflammatory cell infiltration which opposed the mild inflammatory infiltration and moderate pulpal capillary congestion that were seen in our experiment. However, another study conducted by *Maden M. et al.*^30^. reported capillaries congestion in both odontoblastic and central portions of dental pulp capped by MTA for 28 days. This conflict in results could be attributed to that, generally, MTA provides good results in terms of tissue necrosis and hyperemia owing to its preferable characteristics including being nonresorbable, minimally cytotoxic, having high pH setting, reliable cytokine production of cells, high regenerative ability, biocompatibility, antibacterial activity, and an exceptional seal^31^. On the other hand, the undesirable outcomes, reported here and by other previous researches, could be due to poor handling while manipulation or moisture contamination^4^.

In ***group 3***, in BP treated specimens, a thick dentin bridge was formed with a definite predentin layer separating it from the newly formed odontoblast-like cells. This favorable result copes with what was previously recorded by *Liu et al.*^15^ where a heavy dentin bridge was formed after capping the pulp tissues with bioceramic material. This was explained by their in vitro MTT assay that showed that both MTA and iRoot BP materials promoted the proliferation of human dental pulp cells, but BP enhanced the proliferation higher than MTA due to its fine hydrophilic calcium silicate content while bismuth oxide that contained within MTA may reduce its biocompatibility. So, The BP success could be credited to its goodsealing ability, biocompatibility, alkalinity and nontoxicity^5,6^.

Further, *Laurent et al.*^32^ study stated that BP significantly increases the secretion of a key signaling factor for reparative dentinogenesis, transforming growth factor-1(TGF-1), thus favoring pulpotomy success. Besides, BP upregulating the expression levels of odontogenic and focal adhesion molecules was proved by *Zhu et al.*^33^ thus facilitating mesenchymal cells recruitment and subsequent pulp repair. The moderate capillary congestion reported in our experiment could be attributed to the ability of BP to exhibit a higher levels of vascular endothelial growth factor (VEGF) expression and activation from pulpal cells favoring pulpal repair as stated by *Machado et al.*^34^. Finally, BP can also up-regulate the expression of mineralization-related genes^7^ as already proved by our study results of immunohistochemical analysis of DSP expression.

***In group 4***, although TA treated specimens showed dentin bridge formation, irregular pulp calcifications together with radicular pulp necrosis were also seen. The immunohistochemical localization for DSP depicted moderate positive immunoreactivity in the newly formed osteodentin layer. According to literature, no previous study examined TA as a pulp capping material, however, TA was previously added to water used to set MTA and found to positively improve the capping material regarding hydrophilicity, resistance to compression and number of pores^35^. Also, researchers studied the efficacy of TA as an implant coating and found that TA both enhances osteointegration and favorably manipulates the osteoimmunomodulation^36^. This could be attributed to TA ability to favor proliferation and mineralization processes of hard tissue forming cells through activating different signaling pathways including Wnt/b-catenin, Runt-related transcription factor-2 (Runx2), Osterix (Osx), insulin-like growth factor-1 and bone morphogenetic proteins^37^. In addition, other advantageous properties of TA were recorded including being antibacterial and bioactive antioxidant that helps to relieve oxidative stress and prevent the activity of reactive oxygen species (ROS) that are involved in triggering oxidation chain reactions, disrupting typical cellular activity and producing inflammation and tissue death^38^.

However, strikingly, many natural antioxidants, that typically reduce ROS, can also show a negative prooxidant action generating ROS. Although TA has proved to act as a powerful antioxidant against hydroxyl radicals and have a high hydrogen peroxide scavenging effect, it also showed a prooxidant activity, particularly through binding copper ions in chromatin and damaging DNA^39^. In addition, TA displayed an antinutrient action through binding to proteins and dietary ions thus preventing their absorption while digestion^40^. This antioxidant\prooxidant TA activity was found to be concentration dependent. While lower concentrations provide a positive antioxidant effect, higher concentrations demonstrate a prooxidant activity that increase oxidation^41^. This may explain the reason behind the unfavorable results of irregular pulp calcifications and radicular pulp necrosis seen in our TA capped specimens, in which high concentration of pure TA was used for capping, while a more favorable results were seen when TA was just added to water used to set MTA^35^.

Regarding statistical results, the assessment of the thickness of newly formed dentin bridge of different groups revealed a significant difference found between group 1-B and both of group 2, 3 & 4. Also, a significant difference was found between both group 2&3 and group 3&4. This was explained by the study of Zhang et al.^7^ that investigated and compared the effect of iRoot BP with MTA on dental pulp cells. They found that iRoot BP significantly up-regulated the activity of alkaline phosphatase (ALP) and the expression of dentin sialophosphoprotein (DSPP), dentin matrix protein-1 (DMP-1), and osteocalcin (OCN) of pulp cells. These genes are associated with odontoblastic differentiation and mineralization, subsequently, it enhances dentin bridge formation. The difference between group 2&4 was non-significant although group 4 showed a higher score of dentin bridge thickness. This is due to the positive effects of both MTA and TA on hard tissue forming cells in terms of proliferation and mineralization as previously mentioned^28,37^.

Finally, the limitations of our study can be summarized as the reduced dimensions of rat molars, potentially influencing the execution of the surgical procedures involving pulp access and the placement of experimental materials.

## Conclusion

Based on the observations recorded by our study, the following points were concluded:

1. The previous researches that noted that MTA is an effective pulpotomy agent with high success rate are verified.
2. PB proved to be a pulpotomy agent with an exceptional action regarding pulp repair and dentin bridge formation.
3. Although TA provided a noticeable dentin bridge formation, the unfavorable pulp reaction against it requires further studies, using different TA concentrations, to accurately determine the involved mechanisms behind this action.

## Data Availability

All data of this study are available from the corresponding author upon request.
